# PRDM16 as a Multifaceted Pharmacological Target in Kidney Diseases: From Epigenetic Mechanisms to Therapeutic Strategies

**DOI:** 10.3390/ph19091469

**Published:** 2026-09-16

**Authors:** Qiuyu Dai, Shu Rong

**Affiliations:** Department of Nephrology, Shanghai General Hospital, Shanghai Jiao Tong University School of Medicine, Shanghai 200080, China; 18581008966@163.com

**Keywords:** PRDM16, kidney disease, epigenetic therapy, formononetin

## Abstract

PRDM16 is a PR domain-containing epigenetic regulator with intrinsic histone methyltransferase activity and scaffolding interactions with corepressor complexes such as CtBP1/2. Beyond its well-established roles in adipose biology, emerging evidence positions PRDM16 as a central node linking chromatin regulation, metabolic control, and cell survival in the kidney, making it a multimodal pharmacological target. In acute kidney injury, PRDM16 functions as an endogenous protective hub that simultaneously suppresses ferroptosis, apoptosis, and pyroptosis through distinct downstream pathways. In chronic and diabetic kidney disease, TGF-β/Smad-driven silencing or promoter hypermethylation of *PRDM16* disrupts PGC-1α-dependent mitochondrial integrity and podocyte insulin signaling, offering opportunities for epigenetic reactivation strategies and gene therapy. In clear cell renal cell carcinoma, *PRDM16* exhibits a striking duality: epigenetically silenced by CpG island hypermethylation within cancer cells yet upregulated in peritumoral adipocytes to drive a browning-associated, pro-metastatic secretome, defining a unique pharmacological dichotomy that calls for cell-type-selective interventions. This review synthesizes the molecular architecture, isoform diversity, and disease-stage-specific regulation of PRDM16 and evaluates the pharmacological toolset currently available: the expression-inducing natural product formononetin, adenoviral and lentiviral gene delivery systems, and platelet membrane-coated or PLGA-based nanoparticles (PNPs) that enable kidney-targeted delivery. We further discuss key pharmacological challenges, including the need for isoform-selective modulation to avoid oncogenic short-form upregulation, cell-type-specific targeting within the nephron and tumor microenvironment, and the development of pharmacodynamic biomarkers for clinical translation. By integrating mechanistic insights with a drug discovery perspective, this review provides a comprehensive pharmacological framework for targeting PRDM16 to restore renal homeostasis and halt disease progression.

## 1. Background

The kidney is a vital metabolic and excretory organ whose function depends on highly complex and finely tuned intrinsic cellular regulatory networks [1,2,3]. As an increasingly serious public health concern, kidney disease has seen rising global incidence and mortality rates in recent years, affecting approximately 10% of the world’s population [4]. With ongoing advances in research, our understanding of kidney diseases has progressively deepened. These disorders encompass a heterogeneous group of conditions including acute kidney injury (AKI), chronic kidney disease (CKD), and diabetic kidney disease (DKD) [5]. Each type is characterized by distinct pathological mechanisms and requires tailored therapeutic approaches. However, current treatment strategies are predominantly symptomatic and supportive, whereas critical knowledge gaps persist regarding the upstream core regulatory hubs that drive disease initiation and progression. This limitation impairs our understanding of fundamental pathobiological processes and constrains the development of novel, mechanism-based targeted therapies. Consequently, identifying upstream regulatory hubs that are amenable to pharmacological intervention has become a central task in renal drug discovery.

Among the transcriptional regulators that govern kidney cell fate and stress responses, PRDI-BF1 and RIZ homology domain-containing 16 (PRDM16) has attracted particular attention because its intrinsic histone methyltransferase activity and multi-protein scaffolding capacity make it a pharmacologically tractable epigenetic node [6,7,8]. Initially characterized for its essential role in brown adipocyte differentiation and thermogenic regulation [9,10], PRDM16 has since been recognized as an important metabolic–epigenetic integrator with broad physiological functions. Through its PR domain-dependent histone methyltransferase activity and zinc finger structure [11,12], PRDM16 can mediate interactions between proteins, thereby regulating the expression of downstream genes. These actions critically govern fundamental biological processes including cell differentiation, stem cell pool maintenance, energy metabolism, and adaptive responses to tissue stress [13,14,15]. The pharmacological relevance of this pathway is underscored by formononetin, a natural flavonoid compound that enhances *PRDM16* expression and attenuates kidney injury in preclinical models, providing an early chemical starting point for drug development.

Notably, PRDM16 exhibits highly environment-dependent expression and function, varying across tissues, cell types, and disease. This context-dependent duality implies that therapeutic modulation of PRDM16 must be precisely tailored to the disease stage and cell type; otherwise, interventions risk triggering adverse effects such as the oncogenic upregulation of the short isoform. Moreover, emerging evidence has begun to reveal that *PRDM16* is expressed in multiple intrinsic renal cells, including renal tubular epithelial cells and podocytes [16,17], where it participates in early developmental processes and exerts protective effects against renal injury and metabolic dysregulation [18]. Nevertheless, understanding of its precise regulatory networks, mechanisms of action, and clinical translational value within this specific organ remains unclear. Resolving these questions is a prerequisite for designing isoform-selective and cell-type-directed therapies.

This review organizes current knowledge on PRDM16, from its molecular architecture and biochemical functions to its roles in renal pathophysiology, and evaluates the pharmacological toolset that targets this node including the expression-inducing natural product, gene delivery vectors, and kidney-targeted nanocarriers, so as to provide a pharmacological framework for developing precise, mechanism-based therapies for kidney disease.

## 2. Introduction to the PRDM16 Protein

The PRDM protein family constitutes a conserved and functionally versatile class of epigenetic regulators, comprising 17 members in humans (PRDM1–17) [19]. Structurally, all PRDM family members share a hallmark N-terminal PR domain, together with a variable number of C-terminal C2H2-type zinc finger motifs that confer sequence-specific DNA-binding capacity and mediate protein–protein interactions [20,21,22]. Functionally, the family has been implicated in a remarkably broad spectrum of biological processes, ranging from epigenetic control and cell fate specification to embryonic patterning and tumorigenesis [21,23,24,25]. For example, PRDM1 acts as a master transcriptional repressor essential for plasma cell terminal differentiation [26]; PRDM3 is a well-established oncogenic driver in acute myeloid leukemia [27]; and PRDM9 serves as the principal determinant of meiotic recombination in mammals [28]. Despite these shared structural themes, individual family members have evolved highly specialized, often tissue-restricted, physiological roles.

**Figure 1:**
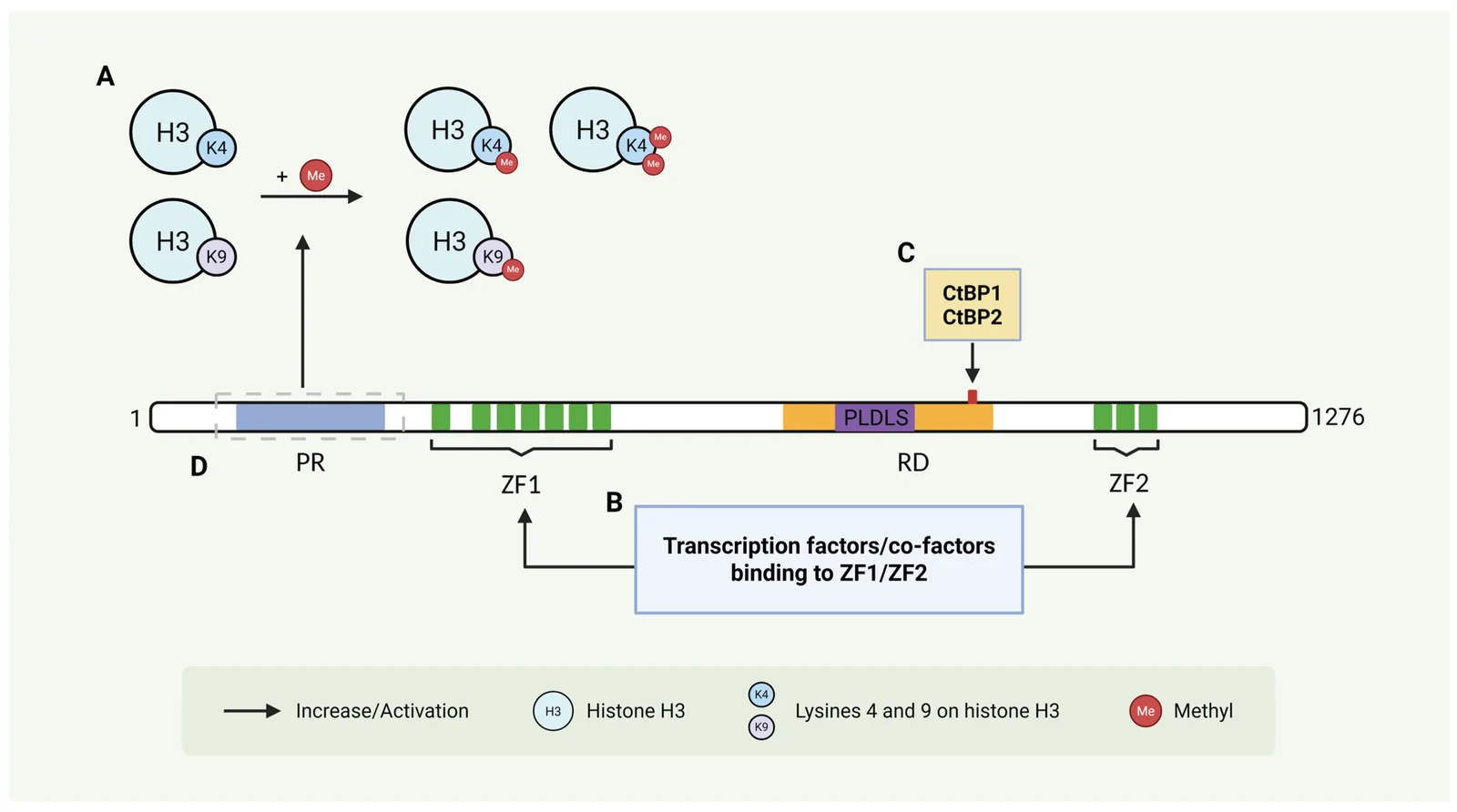
Schematic representation of the major functional domains of PRDM16. The full-length PRDM16 protein (PRDM16-L, 1276 amino acids) contains: (A) a PR domain that possesses histone methyltransferase activity, catalyzing the formation of H3K4me1/2 and H3K9me1 [30,31]; (B) two zinc finger domains (ZF1 at the N-terminus and ZF2 at the C-terminus) that mediate protein–protein and protein–DNA interactions; these domains serve as binding sites for various transcription factors and co-factors, thereby recruiting them to PRDM16 [33]; and (C) a proline-rich repression domain (RD) that harbors a conserved PLDLS motif, which recruits the co-repressors CtBP1/2 to mediate transcriptional repression [32]. (D) The short isoform sPRDM16, generated by alternative promoter usage and splicing, lacks the N-terminal PR domain and has been implicated as an endogenous antagonist of the long isoform [37,38].

Among this family, PRDM16 has emerged as a particularly distinctive member, distinguished by its unique spatiotemporal expression pattern and its multifaceted protective functions across multiple organ systems. PRDM16 is a 1276-amino-acid nuclear protein encoded at 1p36.32 [29]. Its PR domain catalyzes histone H3 lysine methylation [30,31], thereby modulating chromatin accessibility, together with a PLDLS motif that recruits the corepressors CtBP1/2 [32] and two zinc finger clusters that mediate multi-protein assembly [33] (Figure 1). This dual enzymatic and scaffolding architecture renders PRDM16 a pharmacologically tractable epigenetic node. From a drug discovery perspective, the locus generates functionally opposing isoforms through alternative promoter usage: the full-length PRDM16-L retains both the PR domain and zinc fingers and executes metabolic and cytoprotective programs [34,35,36], whereas the short isoform sPRDM16 lacks the PR domain and acts as an oncogenic driver in acute myeloid leukemia and myelodysplastic syndromes [37,38]. This isoform dichotomy demands isoform-selective pharmacological strategies that engage the protective long isoform without inducing the oncogenic short form.

Originally identified as a master regulator of brown adipocyte thermogenesis, where it cooperates with PGC-1α, C/EBPβ and PPARγ to induce UCP1 and drive mitochondrial biogenesis [39,40,41], PRDM16 has since been recognized for its broader protective functions. It maintains stem cell reservoirs in neural, hematopoietic and cardiac tissues [31,42,43,44], guides ventricular morphogenesis by antagonizing TGF-β signaling [45,46], and orchestrates a conserved stress-adaptive response that simultaneously suppresses apoptosis, ferroptosis, NF-κB-driven inflammation and TGF-β-mediated fibrosis [16,47,48]. Its tumor-suppressive dimension is underlined by frequent epigenetic silencing or mutation in melanoma and myeloid malignancies [49]. This protective axis is pharmacologically accessible: the natural product formononetin can upregulate PRDM16 and attenuate kidney injury, while adenoviral and lentiviral gene delivery vectors and kidney-targeted nanocarriers further expand the therapeutic toolkit.

In the kidney, *PRDM16* expression is under tight spatiotemporal control. It is undetectable in the developing metanephros until embryonic day 14.5 [18], coinciding with nephron patterning and maturation, and becomes selectively enriched in proximal tubular epithelial cells [16] and podocytes [17] in the adult organ. This cell-type-restricted distribution aligns directly with PRDM16’s capacity to govern mitochondrial biogenesis, fatty acid oxidation and redox homeostasis, positioning it as a guardian of metabolic fitness in these high-energy-demand populations. At the subcellular level, PRDM16 is a nuclear protein, consistent with its function as a transcriptional regulator that directly binds to chromatin and modulates gene expression through its PR domain-dependent histone methyltransferase activity and zinc finger-mediated protein–protein interactions [11,12,16]. Within the nucleus of renal cells, PRDM16 has been shown to interact with multiple coregulatory partners. These include the transcriptional coactivator PGC-1α, through which it governs mitochondrial biogenesis and fatty acid oxidation [48]; the corepressors CtBP1/2, through which PRDM16 represses the transcription of specific downstream target genes [50]; and the deubiquitinase USP10 and the calcium-binding protein S100A6 [51,52], both of which are involved in PRDM16-mediated cytoprotective signaling cascades in the kidney.

This cell-type-restricted distribution, together with its defined interacting protein network within the nucleus, establishes PRDM16 as a centrally positioned epigenetic hub capable of integrating diverse extracellular signals into coordinated transcriptional outputs in kidney cells, thereby underscoring its potential as a rational therapeutic target for renal diseases, the successful targeting of which will depend on multimodal pharmacological strategies that combine isoform selectivity, cell-type-specific delivery, and context-dependent activity modulation.

## 3. PRDM16 in Kidney Disease: From Developmental Guardian to Context-Dependent Pathological Modulator

### 3.1. PRDM16 in Acute Kidney Injury (AKI)

Acute kidney injury (AKI) is a common clinical syndrome characterized by a rapid decline in kidney function within a short period of time [53]. A leading cause of in-hospital morbidity and mortality, AKI affects approximately 13% of all hospitalized patients and up to 57% of those admitted to intensive care units (ICUs), disproportionately burdening low- and middle-income countries [54,55]. Clinically, AKI can significantly increase short-term mortality, extend hospital stays, and raise medical costs [56]. In the long term, patients face the risk of CKD progression and end-stage renal failure (ESRD); in addition, there is an ongoing rise in cardiovascular events and death [57,58,59]. However, current treatments still mainly focus on supportive care and managing the underlying causes without specific interventions.

In this context, the transcription factor PRDM16 is rapidly emerging as a therapeutically relevant core protective hub in AKI: its expression is rapidly upregulated in renal tubular epithelial cells under stress, subsequently initiating a multi-dimensional cell survival program covering apoptosis, ferroptosis, pyroptosis, and subsequent fibrosis [16,51,52,60]. The pharmacological accessibility of this node has been strongly demonstrated by the successful recapitulation of PRDM16’s protective effect in preclinical models using the expression-inducing natural product, gene delivery vectors, and kidney-targeted nanocarriers.

**Figure 2:**
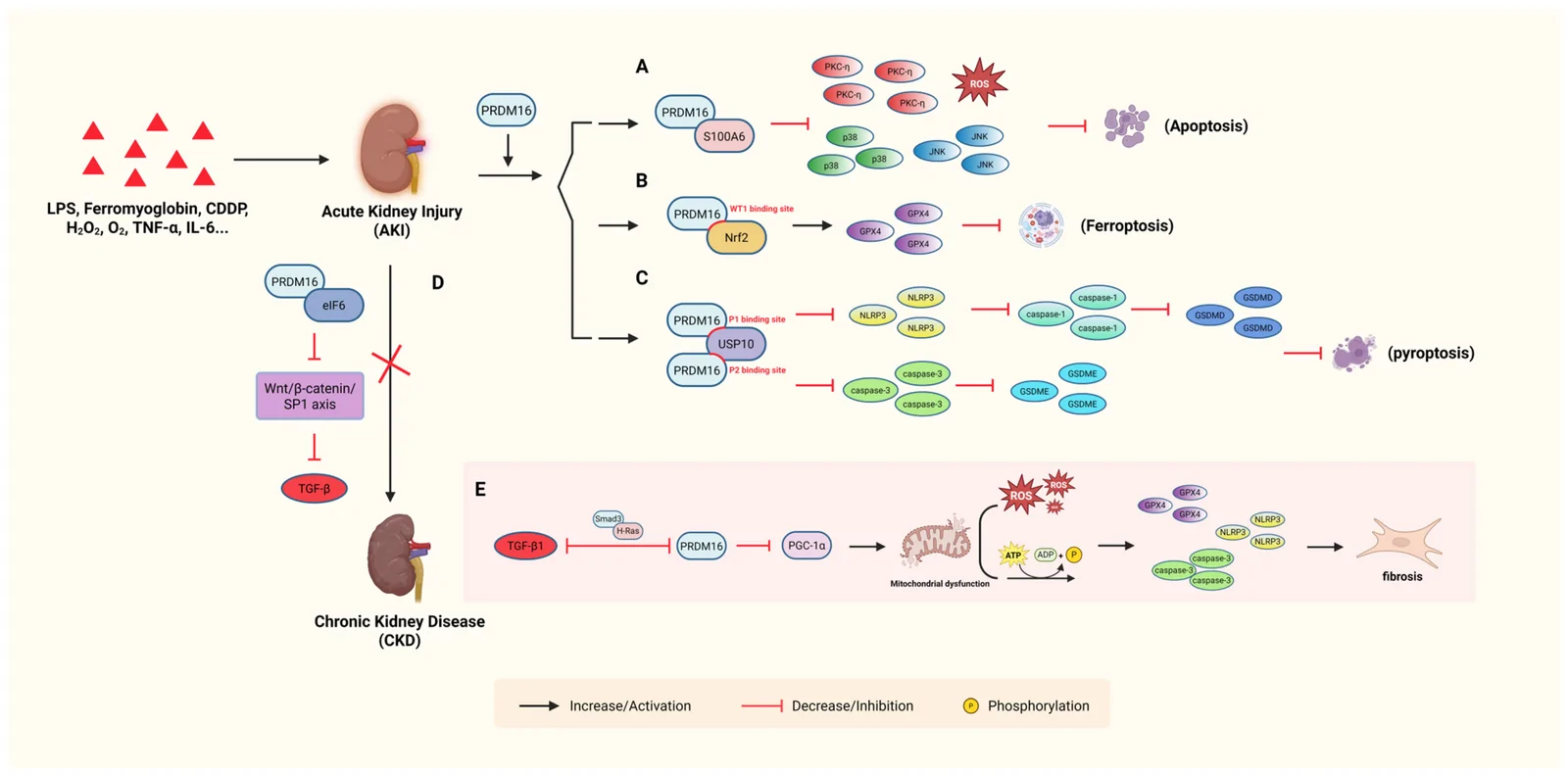
PRDM16 signaling networks in acute kidney injury and chronic kidney disease. (A) Anti-apoptotic pathway in AKI: PRDM16 upregulates S100A6, which inhibits PKC-η, reduces mitochondrial ROS, and blocks p38 MAPK/JNK activation, thereby suppressing tubular epithelial cell apoptosis [51]; (B) Anti-ferroptosis pathway in sepsis-associated AKI: PRDM16 activates the NRF2/GPX4 antioxidant axis, inhibits lipid peroxidation, and protects renal tubular cells from ferroptosis [16]; (C) Anti-pyroptosis pathway in rhabdomyolysis-associated AKI: PRDM16 transcriptionally upregulates USP10, which inhibits both NLRP3/Caspase-1/GSDMD and Caspase-3/GSDME pyroptotic cascades [52]; (D) Blockade of AKI-to-CKD transition: PRDM16 induces eIF6, which suppresses Wnt/β-catenin/SP1 signaling and downregulates TGF-β, thereby limiting fibrotic progression after AKI [60]; (E) Anti-fibrosis pathway in CKD: TGF-β/Smad3 represses PRDM16 transcription; conversely, restored PRDM16 inhibits Smad3 phosphorylation/nuclear translocation and maintains PGC-1α-dependent mitochondrial integrity, forming a negative feedback loop against renal fibrosis [48].

In the classical I/R and cisplatin nephrotoxic AKI models, Li et al. found that the protective effect of PRDM16 was mainly achieved through its carboxyl-terminal domain (CTD) region interacting directly with the calcium-binding protein S100A6, and promoting the expression of S100A6 [51]. Upregulation of S100A6 can inhibit the activity of protein kinase C-η (PKC-η), reduce the production of reactive oxygen species (ROS), and block the overactivation of the downstream p38 MAPK/JNK pathway, thereby building a pharmacologically inducible anti-apoptotic module [51] (Figure 2A). In sepsis-induced AKI, PRDM16 activates the NRF2/GPX4 antioxidant pathway by directly binding to the *WT1* site on *NRF2*, thereby inhibiting lipid peroxidation and ferroptosis and protecting the kidney [16] (Figure 2B). For the rhabdomyolysis-associated AKI, PRDM16 has been shown to directly bind to the promoter region (encompassing the P1 and P2 sites), and then activate the expression of *USP10* [52]. Upregulated USP10 significantly reduces pyroptosis induced by methemoglobin by inhibiting the NLRP3/Caspase-1/GSDMD classical pyroptosis pathway and the Caspase-3/GSDME-mediated alternative pyroptosis pathway, thereby alleviating renal dysfunction [52] (Figure 2C).

Moreover, PRDM16 has shown anti-fibrotic potential in the transition from AKI to CKD. Clinical studies first revealed this association: In renal biopsy specimens from patients with severe obstructive nephropathy (a condition typically characterized by progressive loss of renal function and a tendency towards fibrosis resulting from acute obstruction), *PRDM16* expression was significantly downregulated, accompanied by increased collagen deposition and a marked upregulation of fibrosis markers, suggesting that the absence of PRDM16 may contribute to the malignant progression from AKI to CKD [60]. Based on this, Li et al. discovered through further mechanism studies that PRDM16 binds to a specific site (amino acids 836–847) in the eIF6 promoter, activates eIF6 transcription, inhibits the function of the classical Wnt/β-catenin signaling pathway and its downstream transcription factor SP1, and ultimately leads to the downregulation of key pro-fibrotic factors such as transforming growth factor-β (TGF-β) and connective tissue growth factor (CTGF), thereby delaying the progression of renal tubulointerstitial fibrosis [60] (Figure 2D).

It is worth noting that intervention strategies targeting the PRDM16 pathway have shown promising translational potential. For example, adenovirus vector-mediated PRDM16 overexpression gene therapy [16], as well as the use of PNPs to deliver the expression-inducing natural product formononetin [16,52], can effectively upregulate PRDM16 and its downstream protective pathways in various AKI animal models, significantly alleviating kidney injury and improving functional outcomes.

In conclusion, PRDM16, as a core protective transcription factor activated in the kidney in response to multifactorial insults, regulates key downstream molecules such as USP10, NRF2/GPX4, eIF6, and S100A6 by binding to their promoter regions, constructing a multidimensional, multi-target defense network. This network can specifically inhibit various forms of cell death, including pyroptosis, ferroptosis, and apoptosis, in response to different etiologies while counteracting pro-fibrotic signals. Consequently, it limits the extent of damage during the acute phase of AKI and prevents its malignant progression to CKD during the recovery phase. Moreover, targeting PRDM16 and its related pathways (such as through the application of formononetin or its PNPs) provides a highly promising new strategy for the prevention and treatment of AKI.

### 3.2. PRDM16 in Chronic Kidney Disease (CKD)

Chronic kidney disease (CKD) refers to a clinical condition where structural or functional abnormalities of the kidneys persist for at least three months and affect health [61]. From a pathophysiological perspective, although the causes of CKD are diverse, progressive tissue remodeling and functional loss are common throughout its progression, ultimately leading to renal fibrosis as a shared pathological endpoint [62,63,64]. The TGF-β signaling pathway is the core driver of this process. Due to TGF-β’s broad physiological roles in tissue repair and immune regulation, systemic inhibition often brings significant side effects [65]. Therefore, current research focuses on precision intervention at specific downstream nodes. In line with this trend, recent studies have highlighted a very promising new target—PRDM16.

Clinical data show that in kidney biopsy tissues from patients with IgA nephropathy, *PRDM16* expression levels progressively decrease as renal tubular atrophy and interstitial fibrosis worsen, and they are negatively correlated with blood urea nitrogen levels [48]. At the same time, transcriptome analysis based on the Nephroseq database also confirmed that *PRDM16* expression in the renal tubular interstitium of CKD patients is significantly lower than in normal controls [48]. These data suggest that loss of PRDM16 is a driving event in fibrosis progression, and restoring its expression has therapeutic potential. Unlike previous strategies, PRDM16 is unique in that it is not only a precise downstream effector of TGF-β signaling, but can also form negative feedback regulation on upstream signals [48]. This makes targeting PRDM16 potentially able to block pro-fibrotic signals while correcting the imbalance in the entire pathway. Specifically, in renal tubular epithelial cells, TGF-β1 activates Smad3, which, together with the cofactor H-Ras, directly binds to the promoter region of the *PRDM16* gene, thereby repressing its transcription. Downregulation of PRDM16 further leads to decreased expression of its key downstream target, peroxisome proliferator-activated receptor gamma coactivator 1-alpha (PGC-1α) [48] (Figure 2E). As a core regulator of mitochondrial biogenesis and function, loss of PGC-1α results in severe mitochondrial dysfunction [66,67], characterized by reduced mitochondrial density, disrupted cristae structure, impaired oxygen consumption and ATP production, and weakened fatty acid oxidation (FAO) capacity. The energy metabolism crisis causes lipid accumulation and reactive oxygen species (ROS) buildup in renal tubular epithelial cells and promotes partial EMT processes, leading to the secretion of a large number of pro-fibrotic cytokines [68]. These factors further recruit inflammatory cells and activate myofibroblasts, causing excessive extracellular matrix (ECM) deposition and ultimately driving irreversible renal tubular interstitial fibrosis.

Critically, PRDM16 is not passively regulated by TGF-β; it also exerts negative feedback on the pathway. A recent study showed that overexpression of PRDM16 can inhibit TGF-β–induced phosphorylation of Smad3 and ERK and block the nuclear translocation of p-Smad3, thereby preventing continuous amplification of the TGF-β signaling pathway [48]. This feature of “downstream target pushing back on the upstream pathway” makes PRDM16 an ideal single-target synergy intervention node—pharmacologically activating PRDM16 can not only rescue mitochondrial function but also “turn down” the upstream pro-fibrotic signal switch.

Additionally, CKD is often accompanied by systemic complications such as cachexia, the pathological mechanisms of which involve the browning of white adipose tissue and muscle atrophy. In 2022, a study by Mak et al. found that in CKD model mice, the expression of beige fat cell marker PRDM16 and browning-related genes (*Ucp1*, *Tnfrsf9*, *Tmem26*, *Ppargc1a*, etc.) was significantly upregulated, driving fat browning and muscle wasting. Growth hormone (GH) treatment can reverse this process by downregulating PRDM16 [69]. This difference underscores the importance of developing cell-type-restricted delivery strategies. For example, kidney-targeted nanocarriers could be used to achieve local PRDM16 upregulation while avoiding off-target effects on adipose tissue.

The translational potential of these pathways has already been validated. Lentivirus-mediated, tubule-specific PRDM16 overexpression can reverse mitochondrial damage, lipid deposition, and renal fibrosis induced by TGF-β, unilateral ischemia–reperfusion, and folic acid nephropathy [48]. Combined with studies on formononetin in AKI models and its PNPs, three complementary development strategies are emerging: gene delivery, expression-inducing natural product, and kidney-targeted nanocarriers.

In summary, PRDM16 acts as a negative feedback hub in CKD that can be pharmacologically activated through multiple pathways. By restoring the PRDM16-PGC-1α mitochondrial protection axis while concurrently inhibiting TGF-β/Smad3 signal amplification, targeting PRDM16 can achieve a dual anti-fibrosis effect with a single-target intervention. With the development of tissue-specific delivery technologies, PRDM16 is providing a highly attractive blueprint for precision and combinational therapy of CKD and its systemic complications.

### 3.3. PRDM16 in Diabetic Kidney Disease (DKD)

Diabetic Kidney Disease (DKD) is one of the most severe and common microvascular complications of diabetes, with approximately 30–40% of diabetic patients progressing to DKD [70,71]. Major triggers such as hyperglycemia, accumulation of advanced glycation end-products (AGEs), proteinuria, and lipid metabolism abnormalities drive renal cell injury and adaptive responses [72,73,74,75], ultimately leading to a series of typical structural changes, including glomerular basement membrane thickening, mesangial matrix expansion, and progressive tubulointerstitial fibrosis.

Researchers noticed that, unlike CKD caused by many other etiologies, DKD often exhibits relatively mild tubulointerstitial fibrosis in the early stages of the disease, becoming significant only in the later stages [76]. This unique pathological phenomenon suggests that the kidney may possess an intrinsic protective response mechanism in the early stages of DKD to delay the onset of fibrosis. Relevant clinical data provide direct evidence for this hypothesis. In renal biopsies from patients with DKD, the expression levels of PRDM16 show a clear correlation with the stage of the disease: in patients with early-stage DKD, the levels of PRDM16 protein and mRNA in renal tubular epithelial cells are significantly higher than in normal renal tissue, whereas in patients with advanced DKD, these expression levels decline [17,77]. It is thus evident that the dynamic changes observed in PRDM16 in human DKD kidneys are consistent with its role as an early adaptive protective molecule.

Further research has revealed that PRDM16 is not just a key molecule within this protective network; its functions are also cell-type-specific [17,77]. Rather than performing a single function, it participates in different physiological processes in different renal cell types to address the core survival challenges faced by each cell type in the diabetic environment.

**Figure 3:**
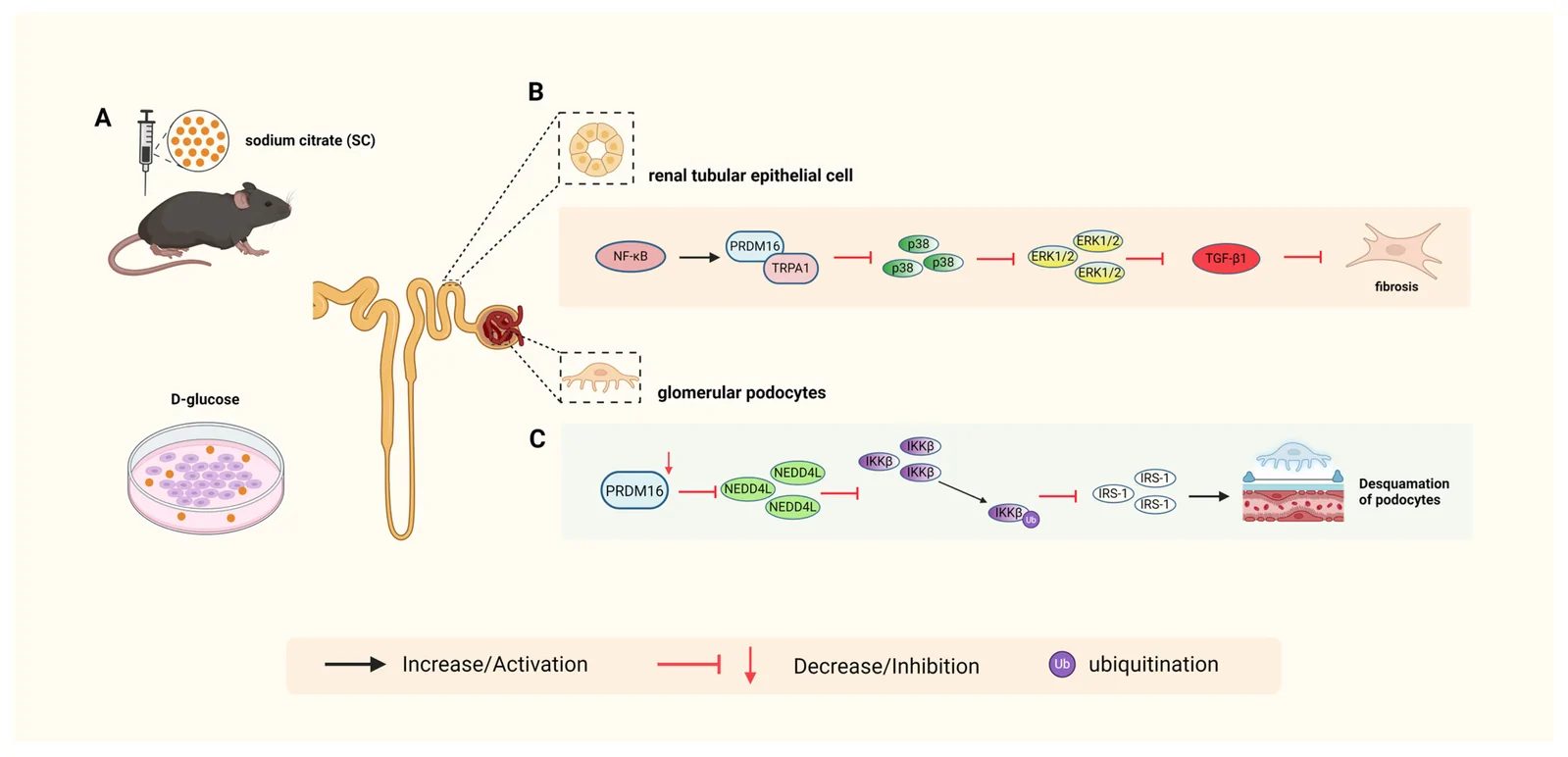
PRDM16 mechanisms in diabetic kidney disease. (A) Schematic illustration of the two primary categories of DKD models: animal models and cell models. (B) In renal tubular epithelial cells: high glucose induces PRDM16 via NF-κB signaling, and then PRDM16 trans-activates TRPA1 to inhibit MAPK/TGF-β1 signaling, thereby suppressing fibrosis [77]. (C) In glomerular podocytes: The loss of PRDM16 leads to reduced transcription of the downstream target gene NEDD4L, thereby inhibiting the ubiquitination and degradation of the kinase IKKβ and increasing its stability. The accumulated IKKβ subsequently promotes the degradation of IRS-1, thereby blocking the insulin receptor signalling pathway within the foot cells [17].

In proximal tubular epithelial cells, PRDM16 plays the role of an adaptive protective factor. In response to high-glucose stimuli, cells actively upregulate the transcription of *PRDM16* by activating the nuclear factor-κB (NF-κB) signaling pathway [78]. The upregulated PRDM16 subsequently initiates a unique negative feedback pathway: it directly binds to and trans-activates the promoter of the transient receptor potential ankyrin 1 (*TRPA1*) gene, thereby inhibiting the activation of the pro-fibrotic mitogen-activated protein kinase (MAPK) signaling pathway (including p38 and ERK1/2), ultimately leading to the downregulation of the core pro-fibrotic factor TGF-β1 [77]. This PRDM16–TRPA1 axis is essentially an endogenous anti-fibrotic circuit that can be pharmacologically amplified: the expression-inducing natural product formononetin, as well as adenovirus-mediated PRDM16 overexpression, can block matrix deposition before the initiation of fibrosis, providing a clear pharmacological entry point for early intervention in DKD [77] (Figure 3B).

In contrast, in glomerular podocytes, PRDM16 functions as a fundamental homeostasis-maintaining factor. In injured podocytes of human DKD patients and diabetic model mice, *PRDM16* expression progressively decreases [17]. Its deficiency leads to reduced transcription of the downstream target gene, the E3 ubiquitin ligase NEDD4L, resulting in impaired ubiquitination-mediated degradation of the kinase IKKβ and increased stability [17]. The accumulated IKKβ then promotes inhibitory serine phosphorylation and degradation of insulin receptor substrate 1 (IRS-1), thereby blocking the insulin receptor signaling pathway within podocytes. Since intact insulin signaling is crucial for maintaining podocyte glucose transport and energy metabolism [79,80], the ‘insulin resistance’ caused by PRDM16 deficiency directly results in an energy metabolism crisis and podocyte injury and loss, driving the progression of glomerular lesions [17] (Figure 3C).

Based on the aforementioned mechanisms, therapeutic strategies targeting PRDM16 need to fully consider cell specificity. For renal tubules, the goal is to enhance their already activated adaptive response. Strategies include adenovirus-mediated PRDM16 overexpression gene therapy or the use of its expression-inducing natural product formononetin to further strengthen the PRDM16-TRPA1 anti-fibrotic axis [77]. For podocytes, the goal is to restore their lost baseline protective functions. Strategies should focus on supplementing *PRDM16* expression through methods such as lentiviral delivery or intervening in its downstream targets (such as supplementing NEDD4L) to mitigate the effects caused by PRDM16 deficiency [17].

Therefore, PRDM16 plays a complex and central dual role in DKD. Its differential expression patterns and functional mechanisms in renal tubules and podocytes vividly illustrate the essence of cell type-specific pathophysiology in the kidney. Future precision therapies targeting PRDM16 must go beyond simply upregulating or downregulating and instead design cell-targeted intervention strategies. This provides a new theoretical framework and a promising translational direction for achieving precision therapy in DKD while simultaneously protecting glomerular and tubular function.

### 3.4. PRDM16 in Renal Clear Cell Carcinoma (ccRCC)

Clear cell renal cell carcinoma (ccRCC) is the most common subtype of renal cell carcinoma, accounting for about 75–80% of all cases [81]. Although progress has been made in targeted drugs and immunotherapy, tumor heterogeneity, drug resistance, and high recurrence rates remain major clinical challenges, urgently requiring the search for new interventional points [82,83]. Recent studies have revealed that the transcription factor PRDM16 exhibits distinct cell-type-specific bidirectional function in ccRCCs, making it an important entry point for understanding disease progression.

In perirenal adipose tissue surrounding the tumor, *PRDM16* expression is upregulated and drives brown remodeling of adipocytes, manifested as a reduction in lipid droplets and marked elevation of brown/beige fat markers such as UCP1 and PGC-1α. This tumor-induced browning profoundly alters the secretory phenotype of adipose tissue. Its conditioned culture medium can induce epithelial–mesenchymal transition in renal tubular epithelial cells and ccRCC cells, upregulating mesenchymal markers such as N-cadherin, thereby enhancing cell migration and invasion capabilities [84]. In contrast, PRDM16 inside tumor cells exerts anticancer functions, but its expression is strictly suppressed by multiple mechanisms. On one hand, high methylation of the promoter CpG island leads to epigenetic silencing [50]; on the other hand, downregulation of the long non-coding RNA *MAGI2-AS3* weakens its sponging of *miR-629-5p*, continuously enhancing the translational inhibition of this miRNA against *PRDM16* mRNA [85]. These mechanisms synergistically maintain low levels of PRDM16, and its low expression is associated with poor patient prognosis. Once expression is restored, PRDM16 can form a complex with the transcriptional co-inhibitor CtBP1/2, specifically suppressing the transcription of tumor target genes such as *SEMA5B*, thereby suppressing tumor growth [50].

This contradictory pattern of microenvironment cancer promotion and intracellular cancer suppression determines that treatment strategies must differentiate cell targets. For perirenal fat, efforts should focus on suppressing PRDM16-mediated abnormal browning and the secretion of epithelial–mesenchymal conversion factors downstream to sever malignant paracrine communication between fat and tumor. For tumor cells, demethylation drugs can be used to undo promoter methylation, or small-molecule agonists can be developed to directly enhance PRDM16 transcriptional activity and protein stability. Tumor-specific overexpression can also be achieved through viral vectors or nanocarriers. Additionally, interventions in ceRNA networks are also feasible, such as overexpressing MAGI2-AS3 or inhibiting *miR-629-5p* to lift post-transcriptional restrictions on PRDM16 and reactivate endogenous tumor suppressor pathways. This multi-level, differentiated intervention strategy is expected to coordinately curb the progression of ccRCC from both the microenvironment and the tumor itself.

### 3.5. PRDM16 in Age-Related Kidney Disease

With the rapid aging of the global population, age-related diseases have become a major public health challenge. In the field of kidney, aging is an independent risk factor for the occurrence and development of CKD [86]. Senescent cells (SnCs) accumulate in the body and create a proinflammatory and pro-fibrotic microenvironment by secreting senescence-associated secretory phenotype (SASP) factors, thereby driving tissue dysfunction and accelerating the process of age-related diseases [87]. Therefore, targeted elimination of senescent cells or intervention of their production mechanisms has become a hot spot in anti-aging therapy. Recent studies have identified the transcription factor PRDM16 as a key molecule linking aging and renal decline.

Studies have shown that the expression of PRDM16 significantly decreases with age in multiple organs, and this decline is closely associated with aging phenotypes [88,89], potentially accelerating the pathological processes of age-related kidney diseases, such as ischemia–reperfusion injury [90]. Conversely, exogenous *PRDM16* gene delivery via lentiviral vectors both in vitro and in vivo can effectively alleviate cellular senescence phenotypes and protect organ function [90]. These findings establish PRDM16 as a key protective transcription factor against aging, and its depletion with aging is an important factor driving aging-related organ dysfunction.

Mechanistically, the anti-aging effect of PRDM16 is closely related to its ability to regulate cellular redox homeostasis and genomic integrity. PRDM16 enhances the glutathione metabolic pathway by directly binding and transcriptionally upregulating the promoter region of glutathione S-transferase Mu 1 (GSTM1) [90]. GSTM1 is an important phase II detoxification enzyme in cells, which catalyzes the conjugation of reduced glutathione (GSH) to electrophilic substrates, thereby neutralizing reactive oxygen species (ROS) and lipid peroxidation products [91,92]. With advancing age, the decline in *PRDM16* expression leads to a corresponding reduction in GSTM1, which in turn impairs the glutathione-dependent antioxidant defense system [90]. This deficiency renders renal tubular epithelial cells vulnerable to oxidative DNA damage and mitochondrial dysfunction, ultimately triggering cellular senescence and reinforcing the pro-inflammatory and pro-fibrotic SASP. In support of this model, restoration of GSTM1 expression in renal tubular epithelial cells has been shown to reverse the cellular senescence and renal aging phenotypes induced by PRDM16 depletion, demonstrating the central role of this pathway [90]. This PRDM16-GSTM1 axis thus provides a new molecular framework for understanding the decline of renal antioxidant capacity during aging and its contribution to the pathogenesis of age-related kidney disease.

Based on these mechanisms, intervention of PRDM16 and its downstream pathways provides a promising strategy for the prevention and treatment of aging-related kidney diseases. The most straightforward strategy is gene replacement therapy, such as the use of modified lentiviral or adeno-associated viral (AAV) vectors, to achieve targeted expression of PRDM16 in kidney cells [90]. In addition, targeting its downstream effector GSTM1, developing small molecule agonists to enhance its enzymatic activity, or supplementing glutathione with prodrugs are feasible ways to exert protection indirectly. These strategies aim to eliminate or prevent the accumulation of senescent cells by augmenting the self-repair and antioxidant capacity of cells from the upstream, which may provide a more fundamental and preventive intervention than direct SnCs.

## 4. Potential Roles of PRDM16 in the Cardiorenal Axis

The cardiorenal axis refers to the complex network through which the heart and kidney interact and regulate each other through multiple pathways such as nerves, endocrine, immunity and metabolism under physiological and pathological conditions. Clinically, cardiorenal syndrome (CRS) often reflects a vicious cycle in which cardiac and renal dysfunction exacerbate each other and progress together, leading to a significant deterioration in the prognosis of patients [93]. Recent studies have found that PRDM16, as a key hub of metabolism and epigenetic regulation, may play an important role in this axis. The dysregulation of its expression and function provides a new molecular perspective to explain how common cardiovascular diseases such as hypertension, coronary heart disease, and heart failure interact with AKI and CKD. In this chapter, the therapeutic potential of PRDM16 as a druggable hub in the heart–kidney interaction network is delineated.

### 4.1. Hypothesized Mechanisms of PRDM16 in AKI Associated with Coronary Heart Disease (CHD)

Patients with coronary heart disease, especially after percutaneous coronary intervention (PCI) or coronary artery bypass grafting (CABG), have a significantly increased risk of AKI [94]. The pathological core of this condition is not an isolated renal event, but rather a classic manifestation of cardio-renal interaction: on the one hand, a reduction in cardiac output directly leads to inadequate renal perfusion; on the other hand, and more crucially, the process of myocardial reperfusion triggers the massive release of inflammatory mediators and reactive oxygen species (ROS), which attack the kidneys via the bloodstream, thereby inducing damage to the renal tubular epithelial cells [95].

**Figure 4:**
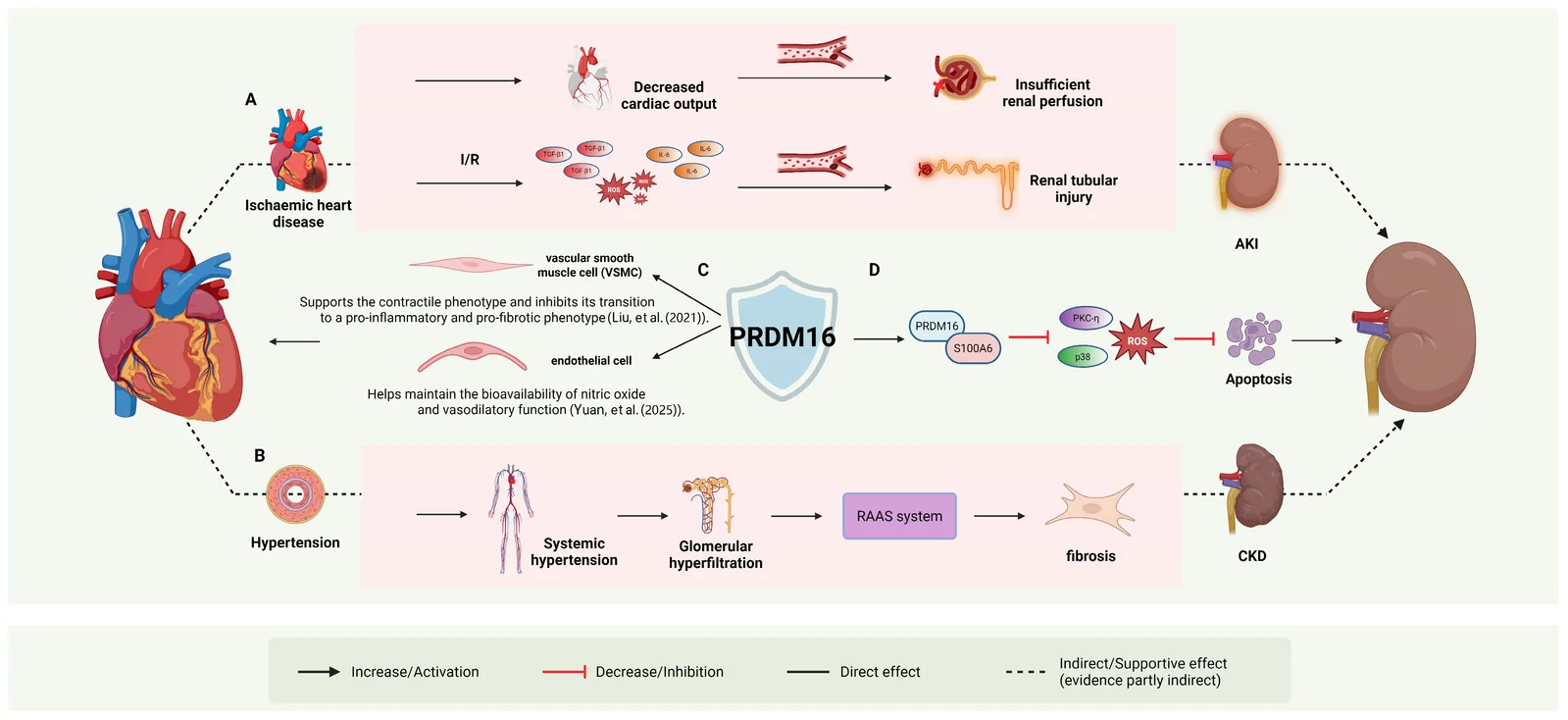
Proposed mechanisms of PRDM16 in the cardiorenal axis [89,90]. (A) Ischaemic heart disease-associated AKI: Decreased cardiac output leads to insufficient renal perfusion. Concurrently, myocardial ischaemia/reperfusion following PCI or CABG triggers the release of ROS, TGF-β, IL-6, and other inflammatory mediators, which directly injure renal tubular epithelial cells, ultimately leading to AKI [94,95]. (B) Hypertension-associated CKD: Systemic hypertension causes glomerular hyperfiltration and RAAS activation, promoting glomerulosclerosis, arteriolar remodeling, and renal interstitial fibrosis, ultimately leading to CKD [96]. (C) Potential beneficial feedback to cardiac function: By preserving VSMC contractile identity and endothelial vasodilatory capacity, PRDM16 may reduce systemic vascular resistance and improve cardiac afterload, potentially ameliorating cardiac dysfunction secondary to hypertension or AKI (hypothesized, not yet experimentally validated in cardiorenal models) [97,98]. (D) Renal protection via S100A6 axis: PRDM16 directly transactivates S100A6, which inhibits PKC-η, lowers mitochondrial ROS, and blocks p38 MAPK/JNK signaling, thereby suppressing tubular epithelial cell apoptosis and improving renal function (demonstrated in renal I/R models) [51].

Against this backdrop of heart–kidney interactions, PRDM16, as an endogenous protective transcription factor in renal tubular epithelial cells, may play a key ‘gatekeeper’ role. Li et al. recently discovered in a classic renal I/R model that *PRDM16* expression is significantly upregulated following ischemia. By directly binding to and activating S100A6, it subsequently inhibits PKC-η activity and reduces mitochondrial ROS production, ultimately blocking the p38 MAPK and JNK signaling pathways, thereby effectively suppressing renal tubular epithelial cell apoptosis [51]. The proposed PRDM16-mediated protective axis in the context of CHD-associated AKI is summarized as a hypothetical model in Figure 4, given that the current evidence is primarily derived from a local renal I/R model rather than direct cardiogenic remote injury models. Although this study utilized a local renal I/R model rather than a cardiogenic remote injury model, the PRDM16-MAPK/JNK protective axis revealed provides direct molecular evidence for understanding how PRDM16 counteracts tubular damage caused by circulating inflammatory factors and oxidative stress. It is therefore reasonable to infer that, amidst the systemic inflammatory and oxidative stress storm triggered by myocardial I/R following PCI or CABG for coronary artery disease, renal PRDM16 may similarly be induced and activated. Through the aforementioned mechanism, it may mitigate the tubular damage caused by remote injury, thereby halting the further progression of the cardio-renal vicious cycle.

Although the current study has tentatively outlined the protective role of PRDM16 in AKI associated with coronary artery disease, numerous key scientific questions remain to be explored in depth. The primary focus is on elucidating the specific mechanisms of inter-organ communication; in particular, determining which specific circulatory factors, following myocardial ischemia–reperfusion, drive the downregulation of *PRDM16* expression or functional impairment in the kidneys. Furthermore, in animal models simulating post-PCI/CABG AKI, it remains to be systematically validated whether PRDM16-targeting interventions (such as the expression-inducing natural product formononetin or gene therapy) can effectively mitigate renal injury while simultaneously improving cardiac function. Future research should prioritize the establishment of combined cardiorenal injury models. By utilizing mice with tubule-specific PRDM16 knockout or overexpression, the necessity and therapeutic potential of PRDM16 in such cardiorenal interactions can be elucidated, thereby truly achieving the therapeutic goal of breaking the cardiorenal vicious cycle.

### 4.2. Potential Role of PRDM16 in Hypertension-Related Chronic Kidney Disease

Hypertension is one of the most common causes of CKD and end-stage renal disease. The mechanism of renal damage is far from simple hemodynamic pressure, but a continuous process involving vascular remodeling, oxidative stress, inflammatory activation and metabolic disorders [96]. Chronic hypertension leads to increased glomerular capillary pressure, triggering endothelial cell damage, podocyte shedding, and stimulating mesangial matrix proliferation, which eventually leads to glomerulosclerosis. At the same time, renal arteriolar hyalinization and intimal thickening (arteriolar remodeling) cause renal parenchymal ischemia, activate the renin–angiotensin–aldosterone system (RAAS), and then drive renal interstitial fibrosis, which is the common end point of irreversible progression of CKD.

PRDM16 plays a fundamental role in maintaining renal vascular homeostasis. In vascular smooth muscle cells (VSMCs), PRDM16 maintains mitochondrial oxidative metabolism by interacting with PGC-1α, which is a key factor supporting their contractile phenotype and inhibiting their transformation to a pro-inflammatory, pro-fibrotic “synthetic” phenotype [97]. In endothelial cells, PRDM16 helps to maintain the bioavailability of nitric oxide and the vasodilation function [98], thereby alleviating intrarenal microcirculation disorders. Thus, PRDM16 constitutes a vascular barrier against hemodynamic injury in hypertension. At the same time, when hypertension persists, the overproduction of reactive oxygen species becomes the core link of kidney injury. At this time, the role of PRDM16 as a key regulator of cellular antioxidant defense is further highlighted. Studies have shown that PRDM16 can directly or indirectly activate antioxidant signaling pathways such as NRF2 [16], and this biological effect effectively provides a basis for renal resistance to reactive oxygen species damage under hypertensive conditions. However, it should be noted that the aforementioned PRDM16-mediated protective mechanisms in the context of hypertensive kidney injury are largely inferred from studies in other disease models or from in vitro experiments, and direct evidence from hypertensive animal models with renal-specific PRDM16 manipulation remains limited. Therefore, the pathway summarized in Figure 4 should be interpreted as a hypothetical framework requiring systematic validation.

Current studies have preliminarily suggested a multi-level protective network of PRDM16 in hypertensive kidney injury. Further studies should focus on the following topics: first, to elucidate the underlying mechanisms and to determine whether and how signaling, such as renin-angiotensin system activation, mechanical stress or specific inflammatory factors, is responsible for PRDM16 downregulation in renal cells in the context of hypertension; secondly, to explore the translational potential of PRDM16 to develop kidney-targeted PRDM16 activation strategies (e.g., small-molecule agonists, nanocarrier delivery systems) and evaluate its renal-protective value in preclinical models beyond traditional antihypertensive therapy to provide new ideas for the intervention of hypertensive nephropathy.

## 5. Discussion

From this review, it is clear that PRDM16 is gradually being redefined from a well-known entity in adipose biology to a regulatory hub of significant value for drug development in kidney disease. Unlike classical renal protective targets that intervene in a single signaling axis, it enables a simultaneous response to multiple pathological stimuli through its triple integration of energy metabolism, modes of cell death and epigenetic status.

The regulation of mitochondrial function represents the most direct manifestation of PRDM16’s function. Whether in the restoration of tubular energy following ischemia, the maintenance of fatty acid oxidation in CKD, or the preservation of insulin sensitivity in podocytes, PRDM16 stabilizes mitochondrial biosynthesis and respiratory function via downstream effector molecules such as PGC-1α. This mechanism responds directly to the expression-inducing natural product: for example, it has been demonstrated that formononetin can activate the PRDM16–PGC-1α axis to alleviate mitochondrial damage, suggesting that targeting a single pathway to rescue upstream energy metabolism is a viable strategy in various renal diseases. More notably, PRDM16’s intervention in cell death mechanisms extends beyond apoptosis; it inhibits pyroptosis via USP10 and ferroptosis via NRF2/GPX4, constituting a cell protection strategy that covers various damage scenarios. This multi-modal cytoprotective capacity suggests that upregulation of *PRDM16* may be effective in acute or chronic kidney injury of various pathological mechanisms without the need to design separate strategies for each form of cell death (Table 1).

What truly sets PRDM16 apart is its role as a negative feedback node in the pathway. In CKD, TGF-β1 suppresses *PRDM16* expression via Smad3 while restoring *PRDM16* expression in turn blocks the phosphorylation and nuclear translocation of Smad3, effectively introducing a self-limiting switch at a central juncture of fibrotic signaling. Unlike unidirectional effectors such as Klotho or NRF2, this bidirectional regulation means that a monotherapy strategy targeting PRDM16 inherently exerts a pathway-correcting effect, both augmenting downstream protective effects and attenuating the sustained amplification of pro-fibrotic signals upstream. This offers a precise solution to the long-standing challenge of translating TGF-β inhibitors into clinical practice, which has been hampered by their toxic side effects.

Of course, in order to develop PRDM16 as a drug target, one must first acknowledge its dual, cell-type-specific behavior. The situation in DKD illustrates this most clearly: proximal tubule cells actively upregulate PRDM16 to counteract TGF-β and oxidative damage, while podocytes become insulin-resistant due to the loss of PRDM16. The tumor microenvironment presents another paradox: while PRDM16 exerts a tumor-suppressive role in clear cell renal cell carcinoma, high *PRDM16* expression in the peri-cancerous adipose tissue actually promotes tumor progression via browning. However, these seemingly intractable functional specificities provide a clear blueprint for the intervention of drug delivery technologies. The renal tubules require reinforcement of their already activated defense programs, podocytes need to re-establish their lost homeostatic functions, and adipose tissue must be prevented from being activated. With the aid of emerging tubule-targeted nanocarriers or podocyte-specific viral vectors, it is entirely feasible to achieve cell-restricted bidirectional regulation within the same kidney, thereby keeping side effects within acceptable limits.

A more fundamental pharmacological challenge, however, lies in the isoform selectivity that we raised earlier but have yet to adequately address. The *PRDM16* locus generates two functionally opposing isoforms through alternative promoter usage: the full-length PRDM16-L, which mediates the protective metabolic and cytoprotective programs discussed throughout this review, and the short isoform sPRDM16, which lacks the PR domain and acts as an oncogenic driver in acute myeloid leukemia and myelodysplastic syndrome. Critically, none of the current intervention strategies (formononetin, adenoviral or lentiviral overexpression, or nanoparticle-based delivery) has been demonstrated to discriminate between these two isoforms. Formononetin, as an expression-inducing natural product, presumably enhances total *PRDM16* expression without distinguishing between the long and short transcripts, as its reported mechanism involves upstream transcriptional activation rather than isoform-specific stabilization. Similarly, viral vector-mediated overexpression typically employs cDNA constructs encoding the full-length PRDM16-L, which in theory should not directly upregulate sPRDM16; however, this strategy carries the inherent risk of unintended activation of the endogenous *sPRDM16* promoter through feedback loops or insertional mutagenesis, a possibility that has not been systematically evaluated in renal contexts. Furthermore, the oncogenic risk of forced PRDM16 overexpression, particularly in the setting of chronic gene therapy, remains largely uncharacterized outside the hematopoietic system. These gaps do not invalidate the therapeutic potential of targeting PRDM16, but they underscore the urgent need for isoform-selective pharmacological tools, such as antibodies or small molecules that specifically stabilize the long isoform or degrade the short isoform, as well as gene-editing approaches like CRISPR-based isoform-specific modulation that could achieve true isoform selectivity. Until such tools become available, the translational framework for PRDM16-targeted therapies must proceed with caution, incorporating rigorous safety monitoring for oncogenic transformation in preclinical large-animal models and, ultimately, in early-phase clinical trials.

The realization of the aforementioned aspirations requires the support of several key pillars. High-precision conditionally engineered mouse models, combined with single-cell and spatial multi-omics approaches, will help establish the direct target gene networks and dynamic response profiles of PRDM16 in different renal cell types; this is an indispensable cornerstone for defining precise intervention windows. At the same time, the development of delivery systems such as those achieving targeting of renal tubules or podocytes through surface modification should be pursued. In parallel with these delivery-focused efforts, the development of gene-editing tools tailored for isoform-selective modulation such as CRISPR-Cas9 systems targeting the *PRDM16* locus, base editors, or antisense oligonucleotides that specifically suppress sPRDM16 while preserving PRDM16-L, represents a complementary and potentially transformative approach. These tools could enable precise manipulation of isoform balance at the genomic or transcriptional level, thereby circumventing the isoform-nonselective nature of current pharmacological interventions. For scenarios where delivery is particularly challenging, small molecules that directly target downstream protective modules (such as NRF2/GPX4 or GSTM1) may serve as an alternative strategy that could enter clinical trials at an earlier stage. With regard to model selection, chronic hypertension models and patient-derived renal organoids will provide an evaluation system that more closely reflects the essence of the disease than existing acute models, enabling long-term observation of the metabolic effects of PRDM16 activation and potential off-target risks.

Ultimately, the value of this preclinical work requires human evidence to be realized. By utilizing established large-scale renal biopsy tissue banks and prospective CKD cohorts to systematically analyze the relationship between the expression levels of PRDM16 and its signaling pathways and the rate of fibrosis progression and renal function decline, it will be possible to identify the patient subgroups most likely to benefit from PRDM16-targeted therapies. At the same time, the development of PRDM16-associated exosome biomarkers in serum or urine is expected to provide liquid biopsy tools for monitoring treatment efficacy and assessing early response during therapy, thereby ensuring that PRDM16-targeted therapies are truly clinically feasible across the entire spectrum, from patient stratification to efficacy tracking.

**Figure 5:**
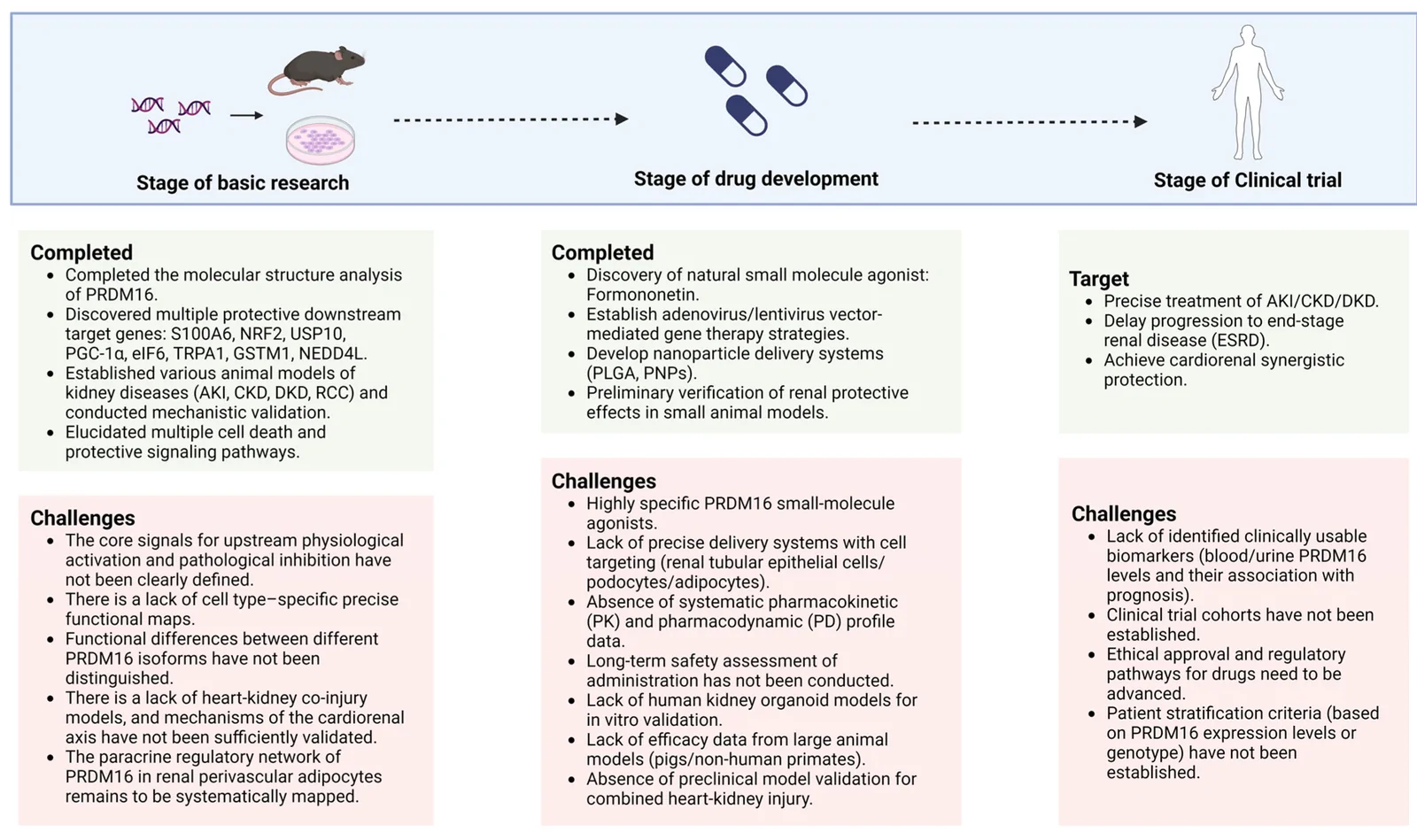
Translational roadmap of PRDM16 research in kidney disease.

Before concluding this article, it is important to acknowledge several limitations that temper the translational conclusions of this review. First, the core renal findings discussed herein, particularly those establishing the PRDM16–PGC-1α mitochondrial axis [50], the PRDM16–TRPA1 anti-fibrotic circuit, the PRDM16–GSTM1 anti-aging pathway, and the PRDM16–NEDD4L–IKKβ axis in podocytes, derive primarily from a limited number of research groups. While these studies are mechanistically rigorous and internally consistent, they have not yet been independently replicated by other laboratories, which is a critical step for establishing the generalizability of these findings. Second, the current evidence base is almost exclusively confined to rodent models of kidney disease, with no large-animal, like porcine, or non-human primate data available to assess the pharmacokinetics, tissue distribution, or long-term safety of PRDM16-targeted interventions in a more translationally relevant setting. Third, clinical data remain scarce; the existing human evidence is limited to observational correlations between *PRDM16* expression levels and disease severity in biopsy specimens, without any interventional studies to demonstrate causality or therapeutic benefit in patients. These limitations do not diminish the scientific merit of the discoveries reviewed here, but they necessitate a measured interpretation of the therapeutic potential. We therefore advocate that the field prioritize independent replication of key findings, systematic evaluation in large-animal models, and the design of well-powered clinical biomarker studies as essential prerequisites before advancing PRDM16-targeted therapies toward clinical translation (Figure 5).

## 6. Methods

### 6.1. Literature Search Strategy

A narrative literature search was conducted in PubMed/Web of Science (WOS) from database inception through August 2026. Search concepts included PRDM16, PRDM protein family, histone methyltransferases, epigenetic regulation, kidney diseases (acute kidney injury, chronic kidney disease, diabetic kidney disease/diabetic nephropathy, renal clear cell carcinoma/clear cell renal cell carcinoma, age-related kidney disease), cardiorenal axis, cardiorenal syndrome, renal fibrosis, PGC-1α, TGF-β signaling, ferroptosis, pyroptosis, apoptosis, formononetin, gene therapy, nanoparticle-based drug delivery, and isoform-selective modulation. Publications were included when they provided directly relevant information on PRDM16 molecular architecture, PR domain function, zinc finger-mediated protein–protein interactions, isoform diversity (PRDM16-L vs. sPRDM16), tissue-specific expression patterns, renal developmental expression, cell-type-specific functions in proximal tubular epithelial cells and podocytes, downstream signaling pathways (NRF2/GPX4, USP10, GSTM1, TRPA1, NEDD4L), or pharmacological intervention strategies including small-molecule compounds, viral gene delivery vectors, and kidney-targeted nanocarriers. Guidelines and consensus statements, systematic reviews and meta-analyses, and original research articles were prioritized. Seminal earlier publications were retained when required to support foundational concepts in PRDM biology, epigenetic regulation, or renal pathophysiology, whereas recent studies were preferentially selected when they addressed emerging mechanistic insights, novel therapeutic modalities, or translational applications. Study selection was narrative and relevance-based; no formal systematic screening, risk-of-bias assessment, or meta-analysis was performed.

### 6.2. Scale for the Assessment of Narrative Review Articles (SANRA)

As a narrative review, this article adheres to the principles of the Scale for the Assessment of Narrative Review Articles (SANRA) [99]. The six SANRA items addressed in this manuscript are (1) justification of the article’s importance for the readership: the growing global burden of kidney disease and the urgent need for mechanism-based therapies establish the clinical relevance of PRDM16 as a pharmacological target; (2) statement of concrete aims: this review systematically synthesizes current knowledge on PRDM16’s molecular architecture, isoform diversity, renal cell-type-specific functions, and evaluates the available pharmacological toolset to provide a comprehensive framework for targeting PRDM16 in kidney diseases; (3) description of the literature search: as detailed above, the search strategy, concepts, and inclusion criteria are explicitly described; (4) referencing: key statements are supported by references to primary literature, with priority given to recent mechanistic studies and high-quality original research; (5) scientific reasoning: the review integrates evidence from molecular, cellular, and preclinical studies to construct a coherent mechanistic framework, incorporating appropriate evidence from diverse experimental models; and (6) appropriate presentation of data: relevant outcome data, including *PRDM16* expression correlations, pathway activities, and therapeutic responses, are presented in the context of their experimental design and limitations.

## 7. Summary

As a multifunctional molecular key, PRDM16 is opening a new door for us to understand the complex mechanism of kidney disease. Its central role in metabolic reprogramming, cell death regulation, and epigenetic integration makes it a common node connecting multiple renal pathophysiological processes. Although the two-sided and complex function of PRDM16 poses great challenges, targeting the PRDM16 pathway holds great potential for precision treatment of kidney diseases. Targeting PRDM16 and its regulatory network is expected to bring new hope for improving the prognosis of millions of patients with kidney diseases worldwide through the continuous deepening of basic research and prudent progress in clinical translation.

## Figures and Tables

**Table 1:** PRDM16-regulated genes and signaling factors in kidney diseases.

Disease Context	Target Gene/Factor	Regulation by PRDM16	Functional Consequence
AKI	S100A6	Induced (direct promoter binding)	Inhibits PKC-η, reduces mitochondrial ROS, blocks p38 MAPK/JNK, suppresses tubular apoptosis [51].
	NRF2	Induced (direct promoter binding)	Inhibits lipid peroxidation and ferroptosis [16].
	USP10	Induced (direct promoter binding)	Inhibits NLRP3/Caspase-1/GSDMD and Caspase-3/GSDME pyroptosis [52].
AKI-to-CKD transition	eIF6	Induced (direct promoter binding)	Suppresses Wnt/β-catenin/SP1, downregulates TGF-β/CTGF, attenuates fibrosis [60].
CKD	PGC-1α	Induced (downstream target)	Maintains mitochondrial biogenesis and fatty acid oxidation, prevents metabolic crisis and fibrosis [48].
DKD	TRPA1	Induced (direct promoter binding)	Inhibits p38/ERK MAPK, downregulates TGF-β1, limits tubulointerstitial fibrosis [77].
	NEDD4L	Induced	Ubiquitinates and degrades IKKβ, preserves IRS-1/insulin signaling, prevents podocyte injury [17].
ccRCC	SEMA5B	Suppressed (via CtBP1/2 complex)	Inhibits tumor growth [50].
Age-related kidney disease	GSTM1	Induced (direct promoter binding)	Enhances glutathione detoxification, reduces oxidative stress and cellular senescence [90].

## Data Availability

No new data were created or analyzed in this study. Data sharing is not applicable to this article.
